# REFRAMING (MY OWN) AGING: SCHOLARLY AND PERSONAL PERSPECTIVES ON PRODUCTIVE AGING

**DOI:** 10.1093/geroni/igad104.0399

**Published:** 2023-12-21

**Authors:** Nancy Morrow-Howell

**Affiliations:** Washington University in St. Louis, St. Louis, Missouri, United States

## Abstract

Advocacy and scholarship grounded in the productive aging paradigm have evolved over 40 years. From an age-drain to an age-as-asset perspective, productive aging scholars have focused on programs and policies to optimize the vital engagement of older people in paid and unpaid work (working, volunteering, caregiving). I have contributed to this body of work and I have evolved with it. I have changed what I study, how I think about later life, and what I think is important for gerontologists to consider. In this presentation, I will review the development of productive aging literature, including tensions that have existed in the scholarship. Themes of ageism and diversity, equity and inclusion have gained prominence in this work; and I will suggest future directions. I will reflect on how my own aging and experiences have shaped my thinking on productive aging.

